# High-Pressure Induced Phase Transitions in High-Entropy Alloys: A Review

**DOI:** 10.3390/e21030239

**Published:** 2019-03-02

**Authors:** Fei Zhang, Hongbo Lou, Benyuan Cheng, Zhidan Zeng, Qiaoshi Zeng

**Affiliations:** 1 Center for High Pressure Science and Technology Advanced Research, Pudong, Shanghai 201203, China; 2 State Key Laboratory for Advanced Metals and Materials, University of Science and Technology Beijing, Beijing 100083, China; 3 China Academy of Engineering Physics, Mianyang 621900, China; 4 Jiangsu Key Laboratory of Advanced Metallic Materials, School of Materials Science and Engineering, Southeast University, Nanjing 211189, China

**Keywords:** high pressure, polymorphic transition, high-entropy alloy

## Abstract

High-entropy alloys (HEAs) as a new class of alloy have been at the cutting edge of advanced metallic materials research in the last decade. With unique chemical and topological structures at the atomic level, HEAs own a combination of extraordinary properties and show potential in widespread applications. However, their phase stability/transition, which is of great scientific and technical importance for materials, has been mainly explored by varying temperature. Recently, pressure as another fundamental and powerful parameter has been introduced to the experimental study of HEAs. Many interesting reversible/irreversible phase transitions that were not expected or otherwise invisible before have been observed by applying high pressure. These recent findings bring new insight into the stability of HEAs, deepens our understanding of HEAs, and open up new avenues towards developing new HEAs. In this paper, we review recent results in various HEAs obtained using in situ static high-pressure synchrotron radiation x-ray techniques and provide some perspectives for future research.

## 1. Introduction

Developing multicomponent metallic alloys with superior properties has played a vital role in the advancement of human civilizations since the Bronze Age. Conventional metallic alloys are usually based on one or two principle elements, such as Fe-, Al-, Mg-, and TiAl- based alloys. Adding alloying elements into the host lattice of the principle elements forming solid solutions has been the major strategy to optimize the microstructures and properties of alloys. However, in the traditional metallurgy, the development of alloys was restricted by the limited solubility of the alloying element in the solvent lattice, which leaves the areas besides the corner of their multicomponent phase diagrams unexplored. Deviation from the phase diagram corner was believed to readily result in the formation of useless, brittle intermetallic compounds. In 2004, Yeh et al. and Cantor et al.’s discoveries of single solid-solution phase alloys formed with multi-principal elements challenged the traditional metallurgy experience and established an exciting new concept for alloy design. By mixing five or more elements with equimolar or near-equimolar ratios, the system could be stabilized in a single phase solid-solution by their maximized configurational entropy [1,2]. Since then, this new class of so-called high-entropy alloys (HEAs) has attracted considerable attention and research interests in the advanced metallic materials community [3,4,5]. Numerous new HEAs have been developed with simple crystal structures such as face-centered cubic (*fcc*) [1,2], body-centered cubic (*bcc*) [1,6,7], and hexagonal close-packing (*hcp*) [8,9,10,11,12]. With unique compositions and atomic structures, HEAs show many interesting properties for potential applications, such as high ductility and strength over a wide temperature range and excellent resistance to both corrosion and wear [13,14,15,16,17,18,19,20,21]. On the other hand, due to their complex composition with a high chemical disorder, many fundamental questions of the HEAs remains challenging to address [3,22,23]. 

One of the critical unsolved questions about HEAs is their phase stability. Many pure elements in the periodic table show rich polymorphic phase transitions [24]. By mapping out the phase diagram (transition paths) with varying temperature and pressure, the phase stability of each structure can be clarified. A famous example is iron. Phase transitions between three different prototype polymorphs with *fcc*, *hcp*, and *bcc* structures were extensively studied in iron [25,26,27]. The *bcc* phase of iron was confirmed to be stable at ambient conditions, while the *fcc* phase is stable at high temperatures and the *hcp* phase is favorable at high pressures. As a combination of multiple elements, HEAs do not simply inherit the structure and properties of their constituent elements as expected with a “linear effect” [28]. Regardless of the various compositions and structures, HEAs are reported to be surprisingly stable over a large temperature range in previous experiments. It is believed that HEAs are thermodynamically stabilized by their high configurational entropy which can largely lower down the Gibbs free energy. Also, the high chemical complexity and packing disorder cause severe local lattice distortion and extremely sluggish atomic diffusion in HEAs, which could further stabilize HEAs kinetically. For instance, the equiatomic CoCrFeMnNi alloy (also named as Cantor’s alloy) [2], is a prototype *fcc*-structured HEA. Extensive studies demonstrate that the Cantor’s alloy can maintain its *fcc* structure from cryogenic temperatures up to its melting temperature without any polymorphic phase transition [13,29,30,31]. 

It has been empirically established that the competition between configuration entropy and enthalpy, the difference between the atomic radius and electronegativity of constituent elements [32,33,34], and also the overall valence electron concentration [35] are a few key thermodynamic parameters for the formation of HEAs. All these parameters are very susceptible to pressure tuning. Actually, pressure is a very powerful tool to tune the atomic/electronic structure of various materials and has been employed to understand materials and to search for novel materials through rich pressure-induced phase transitions in diverse systems, such as pure elements [24], alloys [36,37,38,39,40,41,42], oxides [43,44,45,46], and metallic glass [47,48,49]. Among them, the Ce_3_Al system is of particular interest, where a pressure-induced intermetallic compound and metallic glass to *fcc* solid solution transitions were discovered due to the significant reduction of the difference between both the atomic radii and electronegativity of Ce and Al during compression [42,49]. For HEAs, their synthesis process is closely associated with the competition between intermetallic compounds, metallic glasses, and simple crystalline solid solutions. Therefore, in contrast to the seeming “ultra-stability” during heating or cooling, HEAs might exhibit rich tunable behavior under high pressure. 

Very recently, the structural stability of various HEA systems has been explored using in situ high-pressure synchrotron radiation-based x-ray diffraction techniques. Many interesting polymorphic transitions have been discovered. These results are summarized in Table 1 and are subsequently reviewed in detail. The microstructural and compositional metastability of HEAs was nicely reviewed by Wei et al. [50]. In this paper, we focus on very recent results about phase stability and transitions under high pressure and provide a brief review of the relevant experimental methods, issues, and perspectives for future study.

## 2. Experimental Methods

Diamond anvil cells (DACs) are the most commonly used device to generate high pressure for the in situ studies of materials. DACs are versatile devices for generating pressures up to hundreds of GPa and for combining a full range of in situ measurements [66]. DACs are composed of two opposing diamond anvils that squeeze materials in between them to generate hydrostatic/non-hydrostatic pressure. Since diamonds are transparent to almost the entire electromagnetic spectrum, various in situ electromagnetic radiation detection approaches can be employed to study the structure and properties of samples inside DACs, such as in situ x-ray and neutron diffraction techniques, x-ray emission/absorption spectroscopy, Raman spectroscopy, and Brillouin scattering. Among them, in situ angular dispersive x-ray diffraction (XRD) based on an intense synchrotron radiation x-ray source is the primary technique in the high-pressure structural study of various materials [67]. 

**Figure 1:**
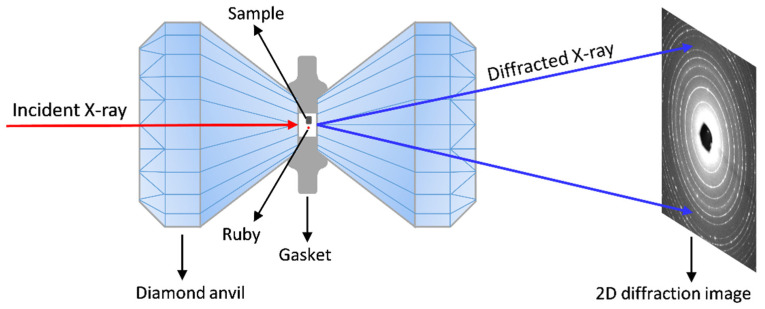
A schematic illustration of the in situ high-pressure synchrotron radiation XRD setup using a diamond anvil cell (DAC).

The size of the DAC anvil culets is typically small with a diameter of approx. 500 μm to 20 μm depending on the target maximum pressure. The sample chamber is a small hole (with a diameter of approx. 1/3 of the culet size and a height of approx. 50 μm to 30 μm) drilled in the gasket indent which is pre-indented by the two anvils. Therefore, only tiny samples with a typical maximum dimension less than tens of microns can be accommodated in the sample chamber in a DAC. Particularly, the sample thickness should be less than the height of the gasket hole during the entire compression process to avoid bridging the anvils (otherwise, it will be uniaxially compressed with large shear stress). Metals with high shear strength such as T301 stainless steel, Re, and W are usually used as the gasket materials for typical high-pressure experiments. Different pressure transmitting mediums (including solid, liquid, and gas) can be selected based on the target pressure range and the required degree of hydrostaticity or operational convenience in the experiment. The pressure in the sample chamber can be determined either by the pressure–volume (*P–V*) equation of state (EOS) of standard materials (e.g., MgO, NaCl, Au, and Pt) using in situ high-pressure XRD [68] or by the ruby fluorescence peak shift excited by an optical laser or x-ray [69]. The standard material or ruby balls should be loaded close to the sample in the sample chamber to minimize the pressure difference. Due to the small sample volume, a high-brightness synchrotron radiation X-ray beam focused down to tens of microns (<20 μm) is required. To go through the two thick diamond anvils (3–5 mm in total) and to cover a large enough range in *d* space with the limited two-theta opening of DACs, a high-energy x-ray is usually required (>20 keV). Using in situ high-pressure synchrotron radiation XRD diffraction coupled with a DAC (a typical experimental setup is shown in Figure 1), researchers have been able to obtain detailed structural information of samples as a function of pressure, including their unit cell parameters, atomic positions, thermal parameters, and even electron density distributions.

## 3. Structural Stability and Evolution of HEAs under High Pressure

Over the last fourteen years, intense effort has been devoted to developing numerous HEAs, which provides hundreds of new alloys for fundamental and applied studies. Recently, the structural stability of some typical HEAs has been investigated using in situ high-pressure synchrotron radiation XRD. Herein, we briefly review these results in separate groups according to their initial crystal structures.

### 3.1. Fcc-Structured HEAs

**Figure 2:**
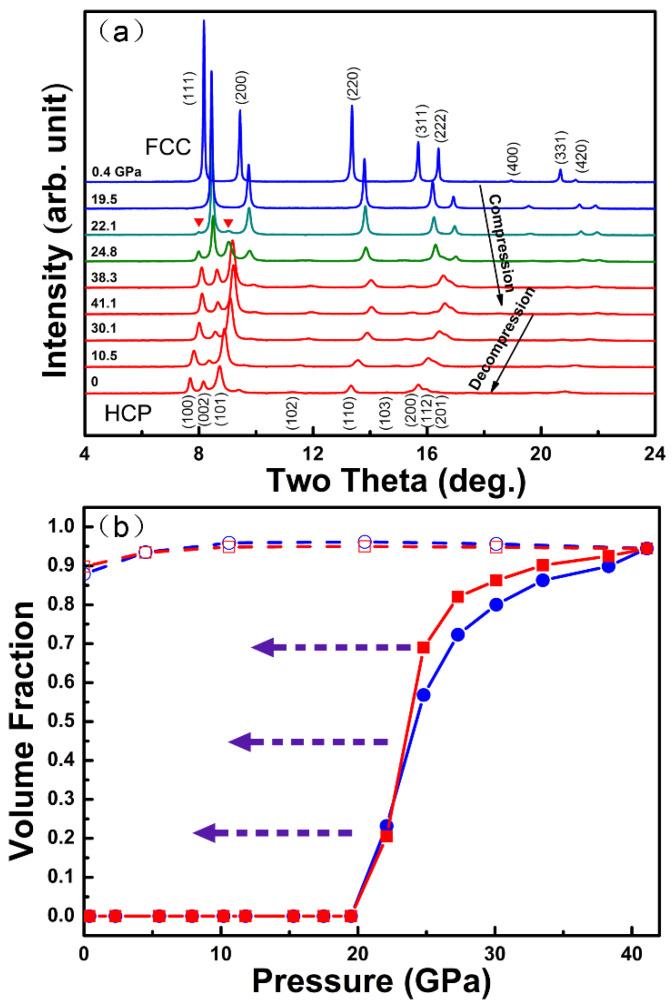
(**a**) The in situ high-pressure XRD patterns of the CoCrFeMnNi HEA under high pressure in a DAC at room temperature [52]: The x-ray wavelength is 0.2952 Å and (**b**) the change of the *hcp* phase volume fraction as a function of pressure during compression (solid symbols) and decompression (open symbols). The volume fractions of the *hcp* phase were calculated based on the peak area changes of the *fcc*-(200) (blue circles) and *hcp*-(101) peaks (red squares) in Figure 2a, which yield consistent results of the volume fractions. During decompression, the volume fraction of the *hcp* almost remains constant, which makes the synthesis of the *hcp*-*fcc* dual-phase composite possible by following a different decompression path, as shown by the dashed arrows [52].

Among the various HEAs, single-phase *fcc*-structured alloy systems tend to show a relatively low yield strength but an excellent ductility and strain hardening capability. The CoCrFeMnNi (Cantor’s alloy) as a prototype *fcc*-structured HEA has attracted the most extensive investigation. Cantor’s alloy shows high structural stability over a broad temperature range at ambient pressures (from extreme low temperature (~3 K) up to its melting temperature) [13,29,30,31]. In contrast, Cantor’s alloy undergoes unexpected polymorphic transformations from *fcc* to *hcp* phases under applied high pressure [51,52,53,54]. Under the best hydrostatic pressure conditions provided by the pressure-medium helium, the *fcc* to *hcp* phase transition starts at approx. 22 GPa but does not fully complete even up to approx. 41 GPa (Figure 2a) [52]. The transition is sluggish and irreversible. The *hcp* phase can be retained during the decompression down to ambient pressure, as shown in Figure 2b. The fabricated *hcp* CoCrFeMnNi HEA can almost maintain its volume fraction during decompression. Therefore, *hcp*-*fcc* dual-phase composites with tunable volume fractions can be readily synthesized by decompression from different maximum pressures between approx. 22 and 41 GPa (Figure 2a) [52]. 

According to previous theoretical simulations, the Gibbs free energy of the *hcp* phase of the CoCrFeMnNi HEA may be smaller than its well-known *fcc* phase at room temperature [70,71]. However, there was no clear experimental evidence to confirm the simulation results. For example, the CoCrFeMnNi HEA samples synthesized by various melt-quenching methods always only show an *fcc* structure, and the irreversibility of the *fcc* to *hcp* phase transition under high pressure also questions the relative stability of the *fcc* and *hcp* phases [51,53,54]. To clarify the phase stability, the synthesized *hcp* phase was further examined using in situ high-temperature XRD measurements at different pressures [52]. During heating at constant pressures, the *hcp* phase transforms back to the *fcc* phase and the critical transition temperatures increase with increasing pressure. Therefore, these results demonstrate that the well-known *fcc* phase of the CoCrFeMnNi HEA is thermodynamically favorable at high temperatures. In contrast, the *hcp* phase is indeed more stable at relatively lower temperatures and higher pressures [52]. 

The pressure-induced *fcc* to *hcp* polymorphic transition has been observed in Cantor’s alloy by independent research groups. However, quite different onset pressures were reported ranging from approx. 7 GPa to even above 49 GPa [51,53,54,55]. Tracy et al. loaded a CoCrFeMnNi HEA sample (annealed at 1200 °C for 24 h) in a DAC with silicone oil as the pressure medium and compressed it up to 54.1 GPa. A sluggish martensitic transformation from the *fcc* to an *hcp* phase was also observed starting at approx. 14 GPa [51]; Huang et al. reported that the *fcc* to *hcp* phase transition occurred at approx. 7.1 GPa in a CoCrFeMnNi HEA sample. The initial sample was processed by a series of heating, cold rolling, and milling and then loaded into a DAC with neon as the pressure medium [54]; Yu et al. prepared a nanograined (approx. 100 nm) CoCrFeMnNi HEA sample by mechanical alloying and a high-pressure sintering process. They compressed the sample with silicone oil as the pressure medium in a DAC up to 31GPa, but no phase transition was observed [56]. Ahmad et al. investigated the structure of Cantor’s alloy up to approx. 49 GPa with neon as the pressure-transmitting medium in a DAC. Surprisingly, no obvious phase transition was observed as well [55]. This experimental inconsistency suggests that the polymorphic phase transition in Cantor’s alloy may be susceptible to the sample and experimental conditions, such as the different sample grain sizes and different pressure mediums used in the experiments above.

**Figure 3:**
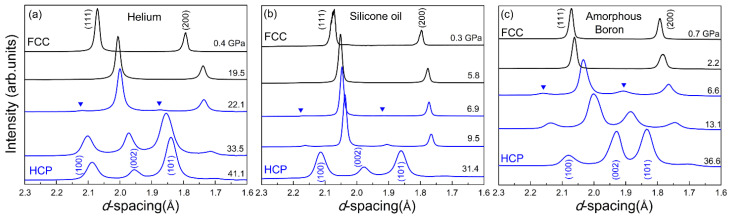
The in situ high-pressure XRD patterns of the CoCrFeMnNi HEA sample with helium (**a**), silicone oil (**b**), and amorphous boron (**c**) as the pressure mediums [53].

To clarify this speculation, Zhang et al. [52] systematically investigated the effect of the non-hydrostaticity of the pressure environment and the grain size of the samples on their pressure-induced phase transitions. The experiments were carefully designed to study only one factor at each time. To address the effect of the pressure environment, they loaded the same sample with three distinct pressure mediums with different degrees of hydrostaticity, such as helium (the most hydrostatic), amorphous boron (the most non-hydrostatic), and silicone oil (quasi-hydrostatic in-between). According to the in situ high-pressure XRD results (as shown in Figure 3), the onset pressures for the *fcc* to *hcp* transition were estimated to be approx. 22 GPa in helium, approx. 2 to 6 GPa in amorphous boron, and approx. 7 GPa in silicone oil. These results demonstrate that the degree of the pressure medium’s hydrostaticity has a positive effect on the onset pressure of the *fcc*-to-*hcp* phase transition in Cantor’s alloy [53]. 

**Figure 4:**
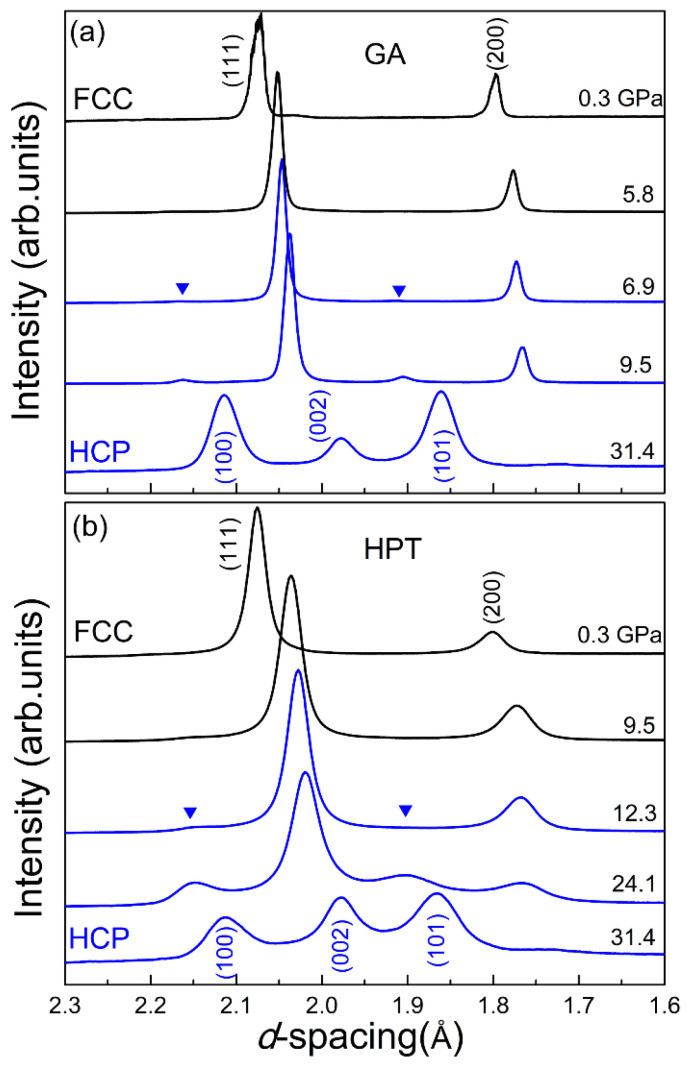
The in situ high-pressure XRD patterns of the gas-atomization (GA) and high-pressure torsion (HPT) CoCrFeMnNi HEA samples loaded in the same DAC with silicone oil as their pressure medium [53].

To study the effect of grain size (an important internal factor) on the pressure-induced phase transition in the CoCrFeMnNi HEA, Zhang et al. [53] loaded two distinct samples into one sample chamber in a symmetric DAC. The two samples were carefully located with equivalent positions to the chamber center to ensure they had identical pressure environments. To highlight the grain size effect, the two selected samples had a huge difference in grain size; one was synthesized by gas-atomization (GA) (approx. 5 μm), and the other was obtained by high-pressure torsion (HPT) (approx. 10 nm). When the two samples were compressed from 0.3 GPa up to 31.4 GPa, both of them showed an *fcc*-to-*hcp* phase transition but with quite different onset pressures (as shown in Figure 4). For the HPT sample with nano-sized grains, the phase transition was observed from approx. 12.3 GPa, while the GA sample with a bigger grain size had a much lower onset pressure of approx. 6.9 GPa. The underlying mechanism is still not clear and calls for further investigation [53]. 

**Figure 5:**
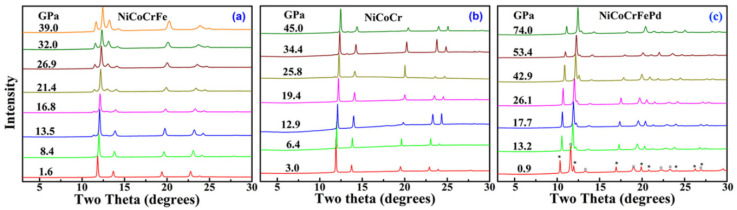
The XRD profiles of CoCrFeNi (**a**), CoCrNi (**b**), and CoCrFeNiPd (**c**) at high pressures: The diffraction peaks marked by symbol * are from the Au pressure standard [57].

The effect of different alloying elements on the pressure-induced polymorphic phase transition was investigated by Zhang et al. [57] in three *fcc*-structured medium-entropy alloys and HEAs (NiCoCr, NiCoCrFe, and NiCoCrFePd). They observed a similar martensitic phase transition from *fcc* to *hcp* in the CoCrFeNi alloy starting at approx. 13.5 GPa, as shown in Figure 5a. This phase transformation was also sluggish and irreversible, as reported in Cantor’s alloy. The volume fraction of the *hcp* phase was only about 36% when the pressure reached 39 GPa. However, with different alloying elements, the NiCoCr and NiCoCrFePd alloys exhibited distinct compression behaviors under high pressure, as shown in Figure 5b,c, respectively. Only a small amount (<5 wt.%) of the *hcp* phase emerged at 34.4 GPa in the NiCoCr alloy, and its amount barely changes with increasing pressure. In the NiCoCrFePd system, as the element Mn in the Cantor’s alloy is replaced by the Pd, no obvious *hcp* phase emerges up to 74 GPa [57]. 

Many of the HEAs have a minor second phase. Ma et al. [58] investigated an equiatomic CoCrCuFeNiPr HEA sample with dual phases (major disordered-*fcc* and minor ordered-*fcc* phases) using in situ synchrotron radiation high-pressure energy-dispersive XRD (EDXRD). They observed a pressure-induced fast ordering transition from approx. 8 GPa to 16.0 GPa followed by a slow transition up to 106.4 GPa. The initially ordered domain in the CoCrCuFeNiPr HEA was believed to act as embryos. With increasing pressure, the embryos grow into the ordered phase [58]. 

### 3.2. Bcc-Structured HEAs

In addition to the *fcc*-structured HEAs, *bcc*-structured alloys are another major member of the HEA family, which include both the chemically disordered A2 and ordered B2 phases. *Bcc*-structured HEAs often exhibit high yield strength in a very high-temperature regime. Therefore, the stability of the *bcc*-structured HEAs is an exciting topic that has been explored extensively at various conditions, recently also by high pressure. 

Ahmad et al. investigated the structural stability of a TiZrHfNb alloy with a disordered *bcc* structure during compression up to 50.8 GPa; no phase transition was found [55]. Yusenko et al. explored another *bcc*-structured Al_2_CoCrFeNi HEA; it had no phase transition up to 60 GPa as well [59]. Guo et al. studied the structural evolution of a superconducting (TaNb)_0.67_(HfZrTi)_0.33_ HEA during compression up to approx. 100 GPa; its *bcc* structure seemed very robust without any detectable structural transition [63]. No phase transition has ever observed in the *bcc*-structured HEAs. Therefore, it seems that the *bcc*-structured HEAs are incredibly stable, and much higher pressure may be needed to induce phase transitions (compared to the *fcc* family). 

**Figure 6:**
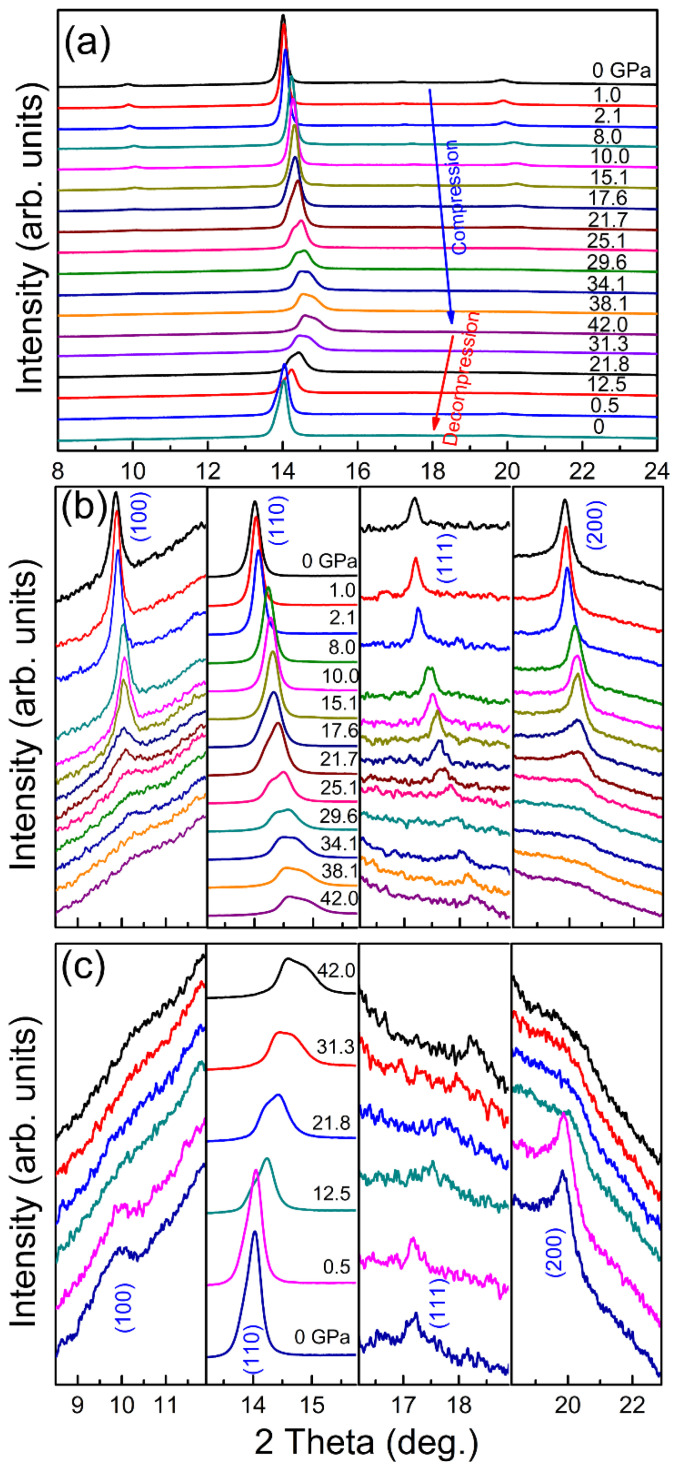
The structural evolution of the AlCoCrFeNi HEA as a function of pressure monitored by in situ high-pressure XRD patterns at room temperature (**a**) and the locally enlarged plot of the XRD patterns for each peak upon compression (**b**) and decompression (**c**) to show more details of the peak shape and width: The x-ray wavelength is 0.4959 Å [61].

To lower down the transition pressure of possible polymorphic phase transitions, Cheng et al. [61] employed a creative strategy to focus on relatively less stable compositions. They chose an equiatomic AlCoCrFeNi HEA and monitored its structural evolution during compression up to 42 GPa. The AlCoCrFeNi alloy had an ordered *bcc*-structure (B2 phase) and was reported to sit in the transition zone between the *fcc* and *bcc* phases, as *x* varies in the Al*_x_*CoCrFeNi HEA system (0 < *x* < 2) [72]. Indeed, they discovered a phase transition from the initial B2 phase to a highly distorted form starting at relatively low pressure of approx. 17.6 GPa, by combining ex situ high-resolution transmission electron microscope (HRTEM) with in situ high-pressure synchrotron radiation XRD data, as shown in Figure 6. Besides the XRD peak splitting, severe peak weakening and broadening occurred during compression, which may have been caused by the significant lattice distortion developed in the sample. Therefore, their work was unable to resolve the atomic structure of the high-pressure phase. Nevertheless, it is the first time that a pressure-induced polymorphism was suggested in a *bcc*-structured HEA [61]. 

**Figure 7:**
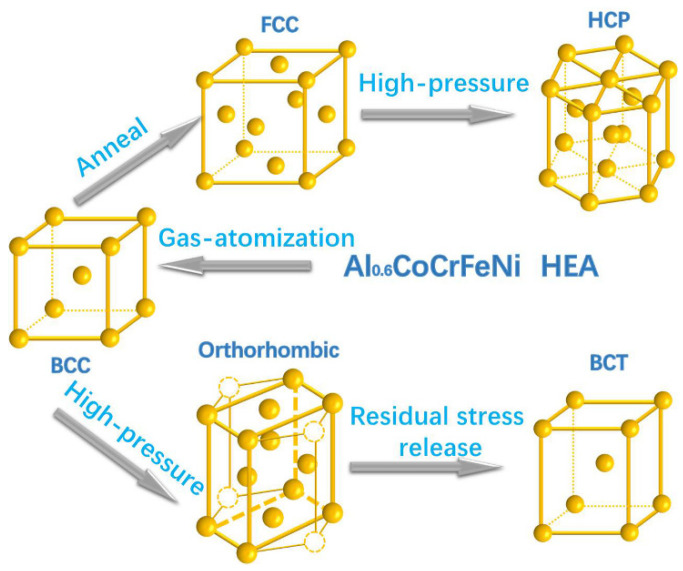
A schematic illustration of the atomic structure for five polymorphs observed in the Al_0.6_CoCrFeNi HEA and the transition paths between them [62].

With slightly lower Al content but still located in the *bcc*-*fcc* transition zone, the Al_0.6_CoCrFeNi HEA was studied by Wang et al. using in situ synchrotron radiation XRD in a DAC with both silicone oil and helium as the pressure-transmitting medium up to approx. 40 GPa [62]. The Al_0.6_CoCrFeNi HEA powders were prepared by the GA method. A single *bcc* phase was obtained with a high quenching rate in the GA process. They revealed a *bcc*-to-orthorhombic phase transition, which started at approx. 10.6 GPa and completed at approx. 21.4 GPa. Interestingly, another body-center-tetragonal (*bct*) phase emerged and coexisted with the high-pressure synthesized orthorhombic phase when the pressure was released. These results indicate that the orthorhombic phase may be metastable at ambient conditions but could be partially maintained due to the possible large energy barrier [62]. Moreover, after annealing at 1000 °C for 2 h, the initial GA *bcc* Al_0.6_CoCrFeNi HEA can transform into a more stable *fcc* phase. During compression, the *fcc* phase of the Al_0.6_CoCrFeNi HEA could completely transform into an *hcp* phase similar to Cantor’s alloy. The samples recovered from high-pressure compression were characterized by transmission electron microscopy (TEM) and further confirmed that all of the five polymorphs could stably/metastably exist at ambient conditions (transition path between them is shown in Figure 7). Severe lattice distortion, which is tunable by high pressure or temperature was suggested to play a crucial role in the formation of various polymorphs and the transition between them in the Al_0.6_CoCrFeNi HEA. These findings suggest that HEAs could behave quite differently from the expectation of a linear combination of its constituent element; they may also exhibit structural flexibility/tunability far beyond that of their solution components [62]. 

### 3.3. Hcp-Structured HEAs

HEAs commonly form with *fcc* or *bcc* structures. Recently, *hcp*-structured HEAs were observed in high-pressure experiments via an *fcc* to *hcp* polymorphic transition and was obtained in the melt-quenched alloys mainly consisting of heavy *hcp* metals, e.g., the CoOsReRu, CoFeReRu and CoReRuV, Ir_0.19_Os_0.22_Re_0.21_Rh_0.20_Ru_0.19_, or rare earth *hcp* elements [8,9,10,11,12]. Ahmad et al. [55] investigated the structural stability of the quarternary equiatomic ReRuCoFe alloy under high pressure. They compressed the sample from 0.9 GPa to 80.4 GPa in a DAC. The unit cell parameters of *a* and *c*, its ratio *a*/*c*, and the sample volume all continuously decreased with increasing pressure, which indicates the *hcp* structure of the ReRuCoFe alloy is stable under compression up to approx. 80 GPa. Yusenko et al. investigated the structural stability of the *hcp* Ir_0.19_Os_0.22_Re_0.21_Rh_0.20_Ru_0.19_ HEA at room temperature during compression up to 45 GPa but also observed no phase transition [64]. 

**Figure 8:**
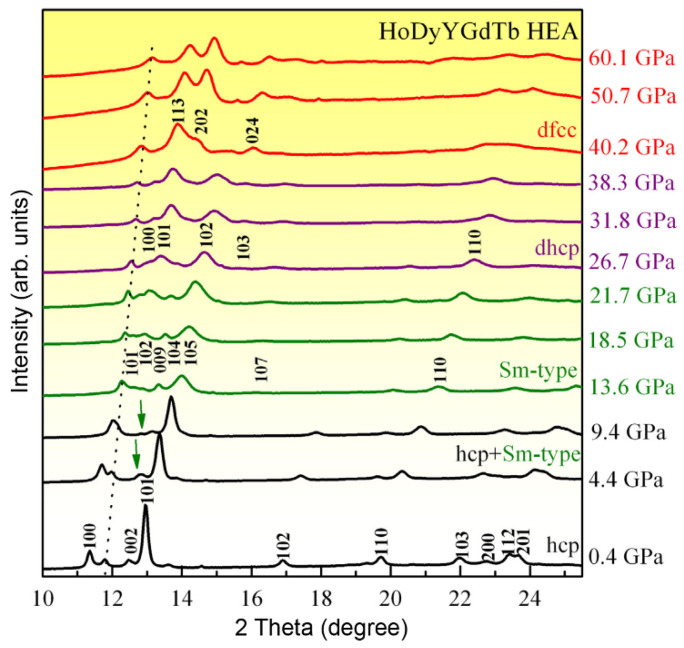
The in situ high-pressure XRD patterns of the HoDyYGdTb HEA during compression [65].

For the *hcp*-structured rare earth HEAs, Yu et al. reported a series of pressure-induced phase transitions in the HoDyYGdTb HEA by in situ XRD measurements in a DAC using synchrotron radiation x-ray. Four polymorphs were observed following a transition sequence of *hcp*→Sm-type→d*hcp*→d*fcc* during compression up to 60.1 GPa (Figure 8), which resembles the rich pressure-induced polymorphic transitions in its constituent elements [65]. The Sm-type phase firstly appeared when the pressure reached 4.4 GPa. At 13.6 GPa, the *hcp* (102) diffraction peak disappeared, indicating the completion of the *hcp* to Sm-type phase transition. With further increasing pressure to 26.7 GPa, the d*hcp* phase emerged and persisted to 38.3 GPa, and then, the d*hcp*-d*fcc* phase transition occurred at 40.2 GPa. 

## 4. Conclusions and Outlooks

HEAs are the focus of advanced metallic alloy research and have been attracting more and more attention over the last decade. Recent studies on HEAs under high pressure have added another dimension to the exploration of HEAs. The exciting findings in HEAs during compression under high pressure deepen our understanding of HEAs, providing a new avenue towards new HEAs development and helpful guidance for applications at extreme conditions. As a new research direction, growing interest in the structure and properties of HEAs under high pressure is expected to continue. Future outlooks are briefly summarized below:

(1) Synergic effect of pressure–composition–temperature. HEAs open up an almost infinite composition space for alloy design. Hundreds of different HEAs have been developed, but so far, only a few of them have been studied under high pressure. Inspired by the existing high-pressure work, more exciting novel phenomena and new structures are expected with broader exploration in more HEAs. Meanwhile, the elusive composition effect on the phase transitions of HEAs remains to be addressed. Besides, combining high pressure with temperature (from cryogenic temperatures up to the melting temperatures) can further clarify the stability of various HEAs and is worth more effort in future research. 

(2) Combing multiple high-pressure techniques for better understanding of HEAs. Over the last few years, the major high-pressure research of HEAs has focused on the crystal structure evolution during compression and decompression. However, we still lack an in-depth understanding of the transformation mechanism. The atomic size ratio, electronegativity difference, valence electron concentration, magnetic states, etc., which are all critical for the formation and transformation of HEAs, have not been systematically explored under high pressure yet. To get more detailed information of the atomic and electronic structure of the multicomponent HEAs under high pressure, besides XRD measurements, more experiments combining other powerful in situ element-sensitive techniques are required, such as in situ high-pressure extended x-ray absorption fine structure (EXAFS), in situ high-pressure x-ray emission spectroscopy (XES), and in situ high-pressure x-ray magnetic circular dichroism (XMCD). 

(3) Involving more variables for high-pressure studies of HEAs. Existing studies have shown that the phase transitions of HEAs are sensitive to shear stress. Therefore, high-pressure torsion (HPT) which generates extreme shear deformation under high pressure could be another powerful technique for HEAs structure tuning with well-controlled shear stress and deformation. In addition, the results reviewed in this paper focus on the static compression of HEAs using DACs. The strain rate effect on the high-pressure behaviors of HEAs has not been extensively investigated. The dynamic compression of HEAs with another dimension of an extremely high strain rate is also worth more exploration.

(4) Properties studies of HEAs using large-volume press. With unique compositions and disordered atomic structures, HEAs show many unusual properties. Under high-pressure compression, HEAs with new structure could be synthesized. Meanwhile, the grain size and defects could also be considerably changed, which could affect their properties as well. Guo et al. measured the superconducting behavior of the (TaNb)_0.67_(HfZrTi)_0.33_ HEA under high pressure. They surprisingly observed extraordinarily robust superconductivity even up to 190.6 GPa [63]. Although the properties of HEAs under high pressure may be interesting, besides the equation of states (EOS) which can be readily measured by in situ XRD in DACs, the vast other properties have not been well-studied. One critical issue of the DAC samples is the requisite tiny sample size. Fortunately, since the critical pressures reported for the polymorphic transitions in HEAs are mostly around 20 GPa or below, a large-volume press (LVP) with approx. 1000 times larger sample volumes than DACs under similar pressure conditions (typically <25 GPa) [73] could be used to synthesize millimeter or centimeter-sized HEAs readily for various properties characterization. Very recently, Yu et al. used a 10-MN double-stage LVP and compressed Cantor’s alloy with a diameter of 1.5 mm and a height of 2 mm to 20 GPa [74]. They synthesized a bulk equiatomic CoCrFeMnNi HEA containing a mixture of *fcc* and *hcp* phases for property characterization. Cantor’s alloy recovered from high-pressure treatment (20 GPa) showed a doubled hardness of the as-cast *fcc* samples because of enhanced dislocations, twins, stacking faults, and the *hcp* laths [74]. 

(5) Theoretical calculations. Recent progress in the experimental discovery of the polymorphic phase transitions in HEAs was first inspired by the finite-temperature ab initio calculation work done by Ma et al., which predicted that the hcp phase in certain magnetic states would be more stable than the *fcc* phase of Cantor’s alloy at room temperature [70]. HEAs with complex compositions are a challenge for theoretical simulations, but it is quite encouraging that much exciting work has been successfully done on HEAs [23,75]. In the high-pressure community, many calculation methods have also been successfully established to handle materials under high pressure [76,77,78]. Theoretical simulations definitely will continue to play a vital part in predicting new phenomena and in interpreting elusive experimental results of HEAs. So far, there is still limited computational calculation works on the high-pressure behaviors of HEAs. However, we believe more exciting works can be expected in HEAs under high pressure by combing advanced experimental tools with simulation methods closely.

The concept of high entropy has been extended into many material systems including high-entropy nitrides, carbides, oxides, and metallic glasses. Therefore, the proposed research above is suitable for other new high-entropy materials as well. 

## Figures and Tables

**Table 1:** A summary of the pressure-induced polymorphic transitions in high-entropy alloys (HEAs) investigated by in situ high-pressure XRD (ME: methanol:ethanol = 4:1 (volume ratio) mixture; MEW: methanol:ethanol:water = 16:3:1 (volume ratio) mixture).

Composition	Initial Structure	Synthesis Method	Grain Size (μm)	Pressure Medium	Max. P (GPa)	Transition P (GPa)	Phase Transition	Ref.
CoCrFeMnNi	*fcc*	Homogenization	/	Silicone oil	54.1	14.7	*fcc-hcp*	[51]
CoCrFeMnNi	*fcc*	Gas-atomization	~ 5	Helium	22.1	41.1	*fcc-hcp*	[52]
CoCrFeMnNi	*fcc*	Gas-atomization	~ 5	Silicone oil	6.9	31.4	*fcc-hcp*	[53]
CoCrFeMnNi	*fcc*	Gas-atomization	~ 5	Amorphous boron	2.2–6.6	36.6	*fcc-hcp*	[53]
CoCrFeMnNi	*fcc*	High pressure tortion	~ 0.01	Silicone oil	12.3	31.4	*fcc-hcp*	[53]
CoCrFeMnNi	*fcc*	Cold rolled	~ 100	Neon	7.1	20	*fcc-hcp*	[54]
CoCrFeMnNi	*fcc*	/	/	Neon	/	48.9	*no*	[55]
CoCrFeMnNi	*fcc*	High pressure sintered	~ 0.1	Silicone oil	/	31	*no*	[56]
CoCrFeCuNi	*fcc*	High pressure sintered	~ 0.1	Silicone oil	/	31	*no*	[56]
NiCoCrFe	*fcc*	Homogenization	/	ME	13.5	39	*fcc-hcp*	[57]
NiCoCr	*fcc*	Homogenization	/	ME	45	45	*fcc-hcp*	[57]
NiCoCrFePd	*fcc*	Homogenization	/	Neon	/	74	*no*	[57]
CoCrCuFeNiPr	*dual fcc*	Milled powder	/	Silicone oil	/	106.4	*disordered-ordered fcc*	[58]
Al_0.3_CoCrFeNi	*fcc*	Annealed	~ 100	Neon	/	61	*no*	[59]
AlCoCrCuFeNi	*fcc+bcc*	As-cast	/	MEW	/	24	*no*	[60]
AlCoCrFeNi	*bcc*	Melt-spun ribbon	/	Silicone oil	/17.6	42	*b*2–*distorted bcc*	[61]
Al_0.6_CoCrFeNi	*bcc*	Gas-atomization	~ 10	Silicone oil/Helium	10.6	40	*bcc-orthorhombic-bct*	[62]
Al_0.6_CoCrFeNi	*fcc*	Gas-atomization + Annealed	~ 5	Silicone oil/Helium	17.5	40	*fcc-hcp*	[62]
TiZrHfNb	*bcc*	/	/	Neon	/	50.8	*no*	[55]
Al_2_CoCrFeNi	*bcc*	Annealed	~ 100	Neon	/	61	*no*	[59]
(TaNb)_0.67_(HfZrTi)_0.33_	*bcc*	As-cast	/	/	/	96	*no*	[63]
ReRuCoFe	*hcp*	/	/	Neon	/	80.4	*no*	[55]
Ir_0.19_Os_0.22_Re_0.21_Rh_0.20_Ru_0.19_	*hcp*	/	/	/	/	45	*no*	[64]
HoDyYGdTb	*hcp*	As-cast	/	Silicone oil	/	60.1	*hcp*→Sm-type→d*hcp*→d*fcc*	[65]

## References

[1] Yeh J.W., Chen S.K., Lin S.J., Gan J.Y., Chin T.S., Shun T.T., Tsau C.H., Chang S.Y. (2004). Nanostructured High-Entropy Alloys with Multiple Principal Elements: Novel Alloy Design Concepts and Outcomes. Adv. Eng. Mater..

[2] Cantor B., Chang I.T.H., Knight P., Vincent A.J.B. (2004). Microstructural development in equiatomic multicomponent alloys. Mater. Sci. Eng. A.

[3] Zhang Y., Zuo T.T., Tang Z., Gao M.C., Dahmen K.A., Liaw P.K., Lu Z.P. (2014). Microstructures and properties of high-entropy alloys. Prog. Mater. Sci..

[4] Lu Z.P., Wang H., Chen M.W., Baker I., Yeh J.W., Liu C.T., Nieh T.G. (2015). An assessment on the future development of high-entropy alloys: Summary from a recent workshop. Intermetallics.

[5] Zhang W.R., Liaw P.K., Zhang Y. (2018). Science and technology in high-entropy alloys. Sci. China Mater..

[6] Senkov O.N., Wilks G.B., Miracle D.B., Chuang C.P., Liaw P.K. (2010). Refractory high-entropy alloys. Intermetallics.

[7] Senkov O.N., Wilks G.B., Scott J.M., Miracle D.B. (2011). Mechanical properties of Nb25Mo25Ta25W25 and V20Nb20Mo20Ta20W20 refractory high entropy alloys. Intermetallics.

[8] Gao M.C., Alman D.E. (2013). Searching for Next Single-Phase High-Entropy Alloy Compositions. Entropy.

[9] Takeuchi A., Amiya K., Wada T., Yubuta K., Zhang W. (2014). High-Entropy Alloys with a Hexagonal Close-Packed Structure Designed by Equi-Atomic Alloy Strategy and Binary Phase Diagrams. JOM.

[10] Feuerbacher M., Heidelmann M., Thomas C. (2015). Hexagonal High-entropy Alloys. Mater. Res. Lett..

[11] Gao M.C., Zhang B., Guo S.M., Qiao J.W., Hawk J.A. (2016). High-Entropy Alloys in Hexagonal Close-Packed Structure. Metall. Mater. Trans. A.

[12] Qiao J.W., Bao M.L., Zhao Y.J., Yang H.J., Wu Y.C., Zhang Y., Hawk J.A., Gao M.C. (2018). Rare-earth high entropy alloys with hexagonal close-packed structure. J. Appl. Phys..

[13] Gludovatz B., Hohenwarter A., Catoor D., Chang E.H., George E.P., Ritchie R.O. (2014). A fracture-resistant high-entropy alloy for cryogenic applications. Science.

[14] Li Z., Pradeep K.G., Deng Y., Raabe D., Tasan C.C. (2016). Metastable high-entropy dual-phase alloys overcome the strength–ductility trade-off. Nature.

[15] He J.Y., Wang H., Huang H.L., Xu X.D., Chen M.W., Wu Y., Liu X.J., Nieh T.G., An K., Lu Z.P. (2016). A precipitation-hardened high-entropy alloy with outstanding tensile properties. Acta Mater..

[16] Huang H., Wu Y., He J., Wang H., Liu X., An K., Wu W., Lu Z. (2017). Phase-Transformation Ductilization of Brittle High-Entropy Alloys via Metastability Engineering. Adv. Mater..

[17] Praveen S., Kim H.S. (2017). High-Entropy Alloys: Potential Candidates for High-Temperature Applications—An Overview. Adv. Eng. Mater..

[18] Liang Y.-J., Wang L., Wen Y., Cheng B., Wu Q., Cao T., Xiao Q., Xue Y., Sha G., Wang Y. (2018). High-content ductile coherent nanoprecipitates achieve ultrastrong high-entropy alloys. Nat. Commun..

[19] Lu C., Niu L., Chen N., Jin K., Yang T., Xiu P., Zhang Y., Gao F., Bei H., Shi S. (2016). Enhancing radiation tolerance by controlling defect mobility and migration pathways in multicomponent single-phase alloys. Nat. Commun..

[20] Zou Y., Ma H., Spolenak R. (2015). Ultrastrong ductile and stable high-entropy alloys at small scales. Nat. Commun..

[21] Chuang M.-H., Tsai M.-H., Wang W.-R., Lin S.-J., Yeh J.-W. (2011). Microstructure and wear behavior of Al_*x*_Co_1.5_CrFeNi_1.5_Ti_*y*_ high-entropy alloys. Acta Mater..

[22] Tsai M.-H., Yeh J.-W. (2014). High-Entropy Alloys: A Critical Review. Mater. Res. Lett..

[23] Miracle D.B., Senkov O.N. (2017). A critical review of high entropy alloys and related concepts. Acta Mater..

[24] Tonkov E.Y., Ponyatovsky E.G. (2004). Phase Transformations of Elements Under High Pressure.

[25] Mao H.K., Bassett W.A., Takahashi T. (1967). Effect of Pressure on Crystal Structure and Lattice Parameters of Iron up to 300 kbar. J. Appl. Phys..

[26] Bassett W.A., Huang E. (1987). Mechanism of the Body-Centered Cubic—Hexagonal Close-Packed Phase Transition in Iron. Science.

[27] Yoo C.S., Akella J., Campbell A.J., Mao H.K., Hemley R.J. (1995). Phase Diagram of Iron by in Situ X-ray Diffraction: Implications for Earth’s Core. Science.

[28] Miracle D.B. (2017). High-Entropy Alloys: A Current Evaluation of Founding Ideas and Core Effects and Exploring “Nonlinear Alloys”. JOM.

[29] Otto F., Dlouhy A., Somsen C., Bei H., Eggeler G., George E.P. (2013). The influences of temperature and microstructure on the tensile properties of a CoCrFeMnNi high-entropy alloy. Acta Mater..

[30] Schneeweiss O., Friák M., Dudová M., Holec D., Šob M., Kriegner D., Holý V., Beran P., George E.P., Neugebauer J. (2017). Magnetic properties of the CrMnFeCoNi high-entropy alloy. Phys. Rev. B.

[31] Wu Z., Bei H., Pharr G.M., George E.P. (2014). Temperature dependence of the mechanical properties of equiatomic solid solution alloys with face-centered cubic crystal structures. Acta Mater..

[32] Zhang Y., Zhou Y.J., Lin J.P., Chen G.L., Liaw P.K. (2008). Solid-Solution Phase Formation Rules for Multi-component Alloys. Adv. Eng. Mater..

[33] Yang X., Zhang Y. (2012). Prediction of high-entropy stabilized solid-solution in multi-component alloys. Mater. Chem. Phys..

[34] Otto F., Yang Y., Bei H., George E.P. (2013). Relative effects of enthalpy and entropy on the phase stability of equiatomic high-entropy alloys. Acta Mater..

[35] Guo S., Ng C., Lu J., Liu C.T. (2011). Effect of valence electron concentration on stability of fcc or bcc phase in high entropy alloys. J. Appl. Phys..

[36] Ming L.C., Manghnani M.H., Katahara K.W. (1981). Investigation of a→w transformation in the Zr-Hf system to 42 GPa. J. Appl. Phys..

[37] Smith D., Joris O.P.J., Sankaran A., Weekes H.E., Bull D.J., Prior T.J., Dye D., Errandonea D., Proctor J.E. (2017). On the high-pressure phase stability and elastic properties of β -titanium alloys. J. Phys. Condens. Matter.

[38] Mao W.L., Campbell A.J., Heinz D.L., Shen G. (2006). Phase relations of Fe–Ni alloys at high pressure and temperature. Phys. Earth Planet. Interiors.

[39] Velisavljevic N., Chesnut G.N. (2007). Direct hcp→bcc structural phase transition observed in titanium alloy at high pressure. Appl. Phys. Lett..

[40] Ahart M., DeVreugd C., Li J., Viehland D., Gehring P.M., Hemley R.J. (2013). X-ray diffraction study of the pressure-induced bcc-to-hcp phase transition in the highly magnetostrictive Fe0.81Ga0.19 alloy. Phys. Rev. B.

[41] Sakai T., Takahashi S., Nishitani N., Mashino I., Ohtani E., Hirao N. (2014). Equation of state of pure iron and Fe_0.9_Ni_0.1_ alloy up to 3Mbar. Phys. Earth Planet. Interiors.

[42] Zeng Q.S., Ding Y., Mao W.L., Luo W., Blomqvist A., Ahuja R., Yang W., Shu J., Sinogeikin S.V., Meng Y. (2009). Substitutional alloy of Ce and Al. Proc. Nat. Acad. Sci. USA.

[43] Dubrovinsky L.S., Saxena S.K., Lazor P., Ahuja R., Eriksson O., Wills J.M., Johansson B. (1997). Experimental and theoretical identification of a new high-pressure phase of silica. Nature.

[44] Hemley R.J., Jephcoat A.P., Mao H.K., Ming L.C., Manghnani M.H. (1988). Pressure-induced amorphization of crystalline silica. Nature.

[45] Bai L., Li Q., Corr S.A., Meng Y., Park C., Sinogeikin S.V., Ko C., Wu J., Shen G. (2015). Pressure-induced phase transitions and metallization in VO_2_. Phys. Rev. B.

[46] Cheng B., Li Q., Zhang H., Liu R., Liu B., Yao Z., Cui T., Liu J., Liu Z., Sundqvist B. (2016). Pressure-induced metallization and amorphization in VO_2_ nanorods. Phys. Rev. B.

[47] Sheng H.W., Liu H.Z., Cheng Y.Q., Wen J., Lee P.L., Luo W.K., Shastri S.D., Ma E. (2007). Polyamorphism in a metallic glass. Nat. Mater..

[48] Zeng Q.-S., Ding Y., Mao W.L., Yang W., Sinogeikin S.V., Shu J., Mao H.-K., Jiang J.Z. (2010). Origin of Pressure-Induced Polyamorphism in Ce_75_Al_25_ Metallic Glass. Phys. Rev. Lett..

[49] Zeng Q., Sheng H., Ding Y., Wang L., Yang W., Jiang J.-Z., Mao W.L., Mao H.-K. (2011). Long-Range Topological Order in Metallic Glass. Science.

[50] Wei S., He F., Tasan C.C. (2018). Metastability in high-entropy alloys: A review. J. Mater. Res..

[51] Tracy C.L., Park S., Rittman D.R., Zinkle S.J., Bei H.B., Lang M., Ewing R.C., Mao W.L. (2017). High pressure synthesis of a hexagonal close-packed phase of the high-entropy alloy CrMnFeCoNi. Nat. Commun..

[52] Zhang F., Wu Y., Lou H.B., Zeng Z.D., Prakapenka V.B., Greenberg E., Ren Y., Yan J.Y., Okasinski J.S., Liu X.J. (2017). Polymorphism in a high-entropy alloy. Nat. Commun..

[53] Zhang F., Lou H., Chen S., Chen X., Zeng Z., Yan J., Zhao W., Wu Y., Lu Z., Zeng Q. (2018). Effects of non-hydrostaticity and grain size on the pressure-induced phase transition of the CoCrFeMnNi high-entropy alloy. J. Appl. Phys..

[54] Huang E.W., Lin C.M., Jain J., Shieh S.R., Wang C.P., Chuang Y.C., Liao Y.-F., Zhang D.Z., Huang T., Lam T.N. (2018). Irreversible phase transformation in a CoCrFeMnNi high entropy alloy under hydrostatic compression. Mater. Today Commun..

[55] Ahmad A.S., Su Y., Liu S.Y., Ståhl K., Wu Y.D., Hui X.D., Ruett U., Gutowski O., Glazyrin K., Liermann H.P. (2017). Structural stability of high entropy alloys under pressure and temperature. J. Appl. Phys..

[56] Yu P.F., Zhang L.J., Cheng H., Zhang H., Ma M.Z., Li Y.C., Li G., Liaw P.K., Liu R.P. (2016). The high-entropy alloys with high hardness and soft magnetic property prepared by mechanical alloying and high-pressure sintering. Intermetallics.

[57] Zhang F.X., Zhao S.J., Jin K., Bei H.B., Popov D., Park C.Y., Neuefeind J.C., Weber W.J., Zhang Y.W. (2017). Pressure-induced fcc to hcp phase transition in Ni-based high entropy solid solution alloys. Appl. Phys. Lett..

[58] Ma Y., Fan J., Zhang L., Zhang M., Cui P., Dong W., Yu P., Li Y., Liaw P.K., Li G. (2018). Pressure-induced ordering phase transition in high-entropy alloy. Intermetallics.

[59] Yusenko K.V., Riva S., Crichton W.A., Spektor K., Bykova E., Pakhomova A., Tudball A., Kupenko I., Rohrbach A., Klemme S. (2018). High-pressure high-temperature tailoring of High Entropy Alloys for extreme environments. J. Alloys Compd..

[60] Li G., Xiao D., Yu P., Zhang L., Liaw P.K., Li Y., Liu R. (2015). Equation of State of an AlCoCrCuFeNi High-Entropy Alloy. JOM.

[61] Cheng B., Zhang F., Lou H., Chen X., Liaw P.K., Yan J., Zeng Z., Ding Y., Zeng Q. (2019). Pressure-induced phase transition in the AlCoCrFeNi high-entropy alloy. Scr. Mater..

[62] Wang L., Zhang F., Nie Z., Wang L., Wang F., Wang B., Zhou S., Xue Y., Cheng B., Lou H. (2019). Abundant polymorphic transitions in the Al_0.6_CoCrFeNi high-entropy alloy. Mater. Today Phys..

[63] Guo J., Wang H., von Rohr F., Wang Z., Cai S., Zhou Y., Yang K., Li A., Jiang S., Wu Q. (2017). Robust zero resistance in a superconducting high-entropy alloy at pressures up to 190 GPa. Proc. Natl. Acad. Sci. USA.

[64] Yusenko K.V., Riva S., Carvalho P.A., Yusenko M.V., Arnaboldi S., Sukhikh A.S., Hanfland M., Gromilov S.A. (2017). First hexagonal close packed high-entropy alloy with outstanding stability under extreme conditions and electrocatalytic activity for methanol oxidation. Scr. Mater..

[65] Yu P.F., Zhang L.J., Ning J.L., Ma M.Z., Zhang X.Y., Li Y.C., Liaw P.K., Li G., Liu R.P. (2017). Pressure-induced phase transitions in HoDyYGdTb high-entropy alloy. Mater. Lett..

[66] Mao H.-K., Chen B., Chen J., Li K., Lin J.-F., Yang W., Zheng H. (2016). Recent advances in high-pressure science and technology. Matter Radiat. Extremes.

[67] Shen G.Y., Mao H.-K. (2017). High-pressure studies with x-rays using diamond anvil cells. Rep. Prog. Phys..

[68] Mao H.-K., Chen X.-J., Ding Y., Li B., Wang L. (2018). Solids, liquids, and gases under high pressure. Rev. Mod. Phys..

[69] Mao H.K., Xu J., Bell P.M. (1986). Calibration of the ruby pressure gauge to 800 kbar under quasi-hydrostatic conditions. J. Geophys. Res..

[70] Ma D., Grabowski B., Körmann F., Neugebauer J., Raabe D. (2015). Ab initio thermodynamics of the CoCrFeMnNi high entropy alloy: Importance of entropy contributions beyond the configurational one. Acta Mater..

[71] Tian F., Varga L.K., Shen J., Vitos L. (2016). Calculating elastic constants in high-entropy alloys using the coherent potential approximation: Current issues and errors. Comput. Mater. Sci..

[72] Wang W.R., Wang W.L., Wang S.C., Tsai Y.C., Lai C.H., Yeh J.W. (2012). Effects of Al addition on the microstructure and mechanical property of AlxCoCrFeNi high-entropy alloys. Intermetallics.

[73] Yamazaki D., Ito E., Yoshino T., Tsujino N., Yoneda A., Guo X., Xu F., Higo Y., Funakoshi K. (2014). Over 1Mbar generation in the Kawai-type multianvil apparatus and its application to compression of (Mg_0.92_Fe_0.08_)SiO_3_ perovskite and stishovite. Phys. Earth Planet. Interiors.

[74] Yu P., Zhang L., Cheng H., Tang H., Fan J., Liaw P.K., Li G., Liu R. (2019). Formation, reverse transformation, and properties of ε-martensite phase in the CoCrFeMnNi high-entropy alloy under high-pressure. J. Alloys Compd..

[75] Tian F. (2017). A Review of Solid-Solution Models of High-Entropy Alloys Based on Ab Initio Calculations. Front. Mater..

[76] Winkler B., Milman V. (2014). Density functional theory based calculations for high pressure research. Cryst. Mater..

[77] Wang Y., Ma Y. (2014). Perspective: Crystal structure prediction at high pressures. J. Chem. Phys..

[78] Zhang L., Wang Y., Lv J., Ma Y. (2017). Materials discovery at high pressures. Nat. Rev. Mater..

